# Interspecies bacterial competition regulates community assembly in the *C. elegans* intestine

**DOI:** 10.1038/s41396-021-00910-4

**Published:** 2021-02-15

**Authors:** Anthony Ortiz, Nicole M. Vega, Christoph Ratzke, Jeff Gore

**Affiliations:** 1 Physics of Living Systems, Department of Physics, Massachusetts Institute of Technology, Cambridge, MA USA; 2 Microbiology Graduate Program, Massachusetts Institute of Technology, Cambridge, MA USA; 3 Present Address: Department of Biology, Emory University, Atlanta, GA USA; 4 Present Address: Interfaculty Institute for Microbiology and Infection Medicine Tübingen (IMIT), Cluster of Excellence ‘CMFI’, University of Tübingen, Tübingen, Germany

**Keywords:** Microbiome, Microbial ecology

## Abstract

From insects to mammals, a large variety of animals hold in their intestines complex bacterial communities that play an important role in health and disease. To further our understanding of how intestinal bacterial communities assemble and function, we study the *C. elegans* microbiota with a bottom-up approach by feeding this nematode with bacterial monocultures as well as mixtures of two to eight bacterial species. We find that bacteria colonizing well in monoculture do not always do well in co-cultures due to interspecies bacterial interactions. Moreover, as community diversity increases, the ability to colonize the worm gut in monoculture becomes less important than interspecies interactions for determining community assembly. To explore the role of host–microbe adaptation, we compare bacteria isolated from *C. elegans* intestines and non-native isolates, and we find that the success of colonization is determined more by a species’ taxonomy than by the isolation source. Lastly, by comparing the assembled microbiotas in two *C. elegans* mutants, we find that innate immunity via the p38 MAPK pathway decreases bacterial abundances yet has little influence on microbiota composition. These results highlight that bacterial interspecies interactions, more so than host–microbe adaptation or gut environmental filtering, play a dominant role in the assembly of the *C. elegans* microbiota.

## Introduction

Bacterial communities are found almost everywhere in nature [1]. Among the many ecosystems in which bacterial communities play a fundamental role, the animal digestive tract is one of remarkable importance [2, 3]. These large, complex, and highly organized bacterial consortia [4] can degrade food and deliver nutrients to their host [5], protect against invading pathogens [6, 7], and even produce neurotransmitters that affect host behavior [8].

**Fig. 1:**
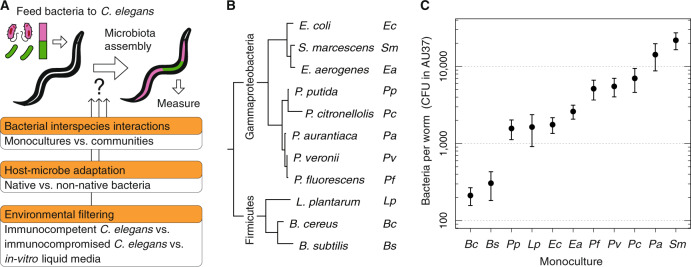
Different bacterial species reach widely different population sizes in *C. elegans* gut. **A** Diagram of the *C. elegans* microbiota assembly and the three biological forces (orange) that might influence this process and that we study in this article. To construct and measure simple microbiotas in *C. elegans*, a defined number of bacterial species are fed in liquid culture to a same-age adult population of *C. elegans* previously sterilized with antibiotics. The liquid feeding substrate is restored every day to maintain equal bacterial concentrations during the 4 days of colonization. Afterwards, worms are mechanically disrupted in batches of ~20, and counts of colony forming units (CFU) are used to determine bacterial population sizes in the worm gut. **B** Phylogenetic tree from full-length 16S rRNA gene sequences of the 11 non-native bacterial species used to colonize the gut of *C. elegans*. **C** Bacterial population sizes in monoculture colonization of immunocompromised *C. elegans* (AU37) span two orders of magnitude. These population sizes reflect the inherent abilities of bacteria to colonize the worm intestine environment. Points are the average of eight or more biological replicates, and error bars are the standard error of the mean (s.e.m.).

Despite considerable efforts toward elucidating the composition and function of these intestinal bacterial communities [9, 10], the rules that govern their assembly are still not fully understood [11]. Recent studies have taken advantage of animal model systems, such as mice [12], zebrafish [13, 14], honey bees [15], flies [16, 17], and worms [18], to experimentally address the composition and assembly of simpler gut microbiotas [19]. A recurrent explanation for the assembly of these communities is that the gut can strongly filter the bacterial colonizers and select for a core microbiota [20, 21]. If such environmental filtering is sustained over evolutionary timescales, an adaptation between hosts and microbes can occur and a symbiosis can develop [22], but not all associations between hosts and microbes are indicative of adaptation or co-evolution [23]. The competition assays via co-culturing microbes [24] and the bottom-up assembly of microbiotas [25] that are possible in animal model systems provide an opportunity to test which forces influence microbiota assembly (Fig. 1A).

The nematode *C*. *elegans* is a good model system to study gut microbiota assembly [26]. Multiple human pathogens are also pathogens for *C. elegans* [26, 27], and the longevity [28] and reproduction [29, 30] of this worm are linked to its microbiota. Furthermore, the *C. elegans* gut environment filters the larger bacterial pool found in natural feeding substrates, leading to the assembly of a core microbiota [31, 32]. Such environmental filtering can occur via behavioral food avoidance [33], ingestion rates [34, 35], and host mucins [36], among other factors [7, 26]. Recent reports have suggested that the worm’s innate immunity [37, 38] and microbe–microbe interactions [39] play a dominant role in the *C. elegans* microbiota assembly, but an experimental comparison of the many forces in play is still lacking.

In this study, we colonized *C. elegans* with simple microbiotas to determine the effect that bacterial interspecies interactions, host–microbe adaptation, and environmental filtering have on the underlying assembly process (Fig. 1A). We found that the ability of a bacterial species to colonize the worm gut in monoculture was often inadequate for predicting the relative abundances of two-, three-, and eight-species microbiotas. Additionally, in experiments with bacteria not isolated from *C. elegans* (non-native), we found that the fractional abundance of two-species microbiotas can be used to predict the composition of three-species microbiotas, indicating that assembly rules based on pairwise interactions [40] can provide insight into the composition of gut microbiota communities. Finally, *C. elegans* and its feeding substrate can reach different stable states, and the acidic pH of the worm gut may be a component of the environmental filtering by this host during community assembly. With this, we advance our understanding of the polymicrobial colonization of the *C. elegans* gut and provide insight into bacterial community assembly within a host.

## Materials and methods

### Nematode culture

Nematodes were grown, maintained, and manipulated with standard techniques [35, 41]. We utilized the *C. elegans* strains N2 (wild type), AU37 [*glp-4(bn2)* I; *sek-1(km4)* X], and SS104 [*glp-4*(*bn2*) I]. Worm strains were provided by the Caenorhabditis Genetic Center, which is funded by NIH Office of Research Infrastructure Programs (P40 OD010440). Synchronized *C. elegans* cultures were obtained using standard protocols [35, 41]

### Bacteria

Non-native bacteria were obtained from ATCC: *Bacillus subtilis* (ATCC 23857) (*Bs*)*, Enterobacter aerogenes* (ATCC 13048) (*Ea*), *Lactobacillus plantarum* (ATCC 8014) (*Lp*), *Pseudomonas aurantiaca* (*Pseudomonas chlororaphis* subsp. *aurantiaca*) (ATCC 33663) (*Pa*), *Pseudomonas citronellolis* (ATCC 13674) (*Pc*), *Pseudomonas fluorescens* (ATCC 13525) (*Pf*), *Pseudomonas putida* (ATCC 12633) (*Pp*), *Pseudomonas veronii* (ATCC 700474) (*Pv*), and *Serratia marcescens* (ATCC 13880) (*Sm*). *Bacillus cereus* (*Bc*) was obtained from Ward’s Scientific Catalog. *Escherichia coli* MC4100 (CGSC #6152) (*Ec*) was obtained from the *E. coli* Genetic Stock Center.

The microbiota strains native to *C. elegans* were isolated by growing *C. elegans* N2 for 1 week on individual types of rotten organic material (apples, celery, almonds, and parsnip), followed by washing and sterilizing the worms on the outside, grinding the worms, and plating the resulting bacterial suspension on agar plates (Supplementary Information). The species identity was analyzed by 16S Sanger sequencing (Genewiz, South Plainfield, NJ, USA).

### Bacterial colonization of *C. elegans*

To construct cultures to feed *C. elegans*, bacterial strains were grown to saturation (24 h, 30 °C, 2 mL LB [Difco]), and then washed in S medium [41] and resuspended in 1% v/v Axenic Medium diluted in S medium (1% AXN). Undiluted Axenic Medium was prepared by autoclaving 3 g yeast extract and 3 g soy peptone (Bacto) in 90 ml water, and subsequently adding with sterile technique 1 g dextrose, 200 µl cholesterol (5 mg/ml in ethanol), and 10 ml of 0.5% w/v hemoglobin (Sigma-Aldrich) in 1 mM NaOH. The bacterial cultures were standardized to ~10^8^ CFU/ml based on CFU counts.

Germ-free adult worms were resuspended in 1% AXN to a concentration of ~1000 worms/mL. Aliquots of 120 µL (~100 worms) were transferred into 96-deep-well culture plates (1 mL well volume, Eppendorf). Bacterial suspensions were added to reach a concentration of ~10^7^ CFU/mL per bacterial strain in all feeding experiments. Plates were covered with Breathe-Easy (Sigma-Aldrich) sealing membranes and incubated for 4 days with shaking at 400 RPM at 25 °C. Every day the worm samples were washed and the bacteria were replenished. Samples were washed with a liquid handler (VIAFLO 96, Integra) by adding 500 µl of M9 Buffer  [41] + 0.1% v/v Triton X-100 (Tx), pipetting 10 times, and removing the supernatant after worms precipitated. The worms were then transferred to new 96-deep-well plates to leave behind possible biofilms, and then washed in the same way two more times with 1% AXN. Fresh bacterial cultures were added as previously described.

### Mechanical disruption of worms and quantification of bacteria

The worm samples were washed to remove most external bacteria and then incubated in 100 µL S medium + 2X heat-killed OP50 at 25 °C for 1 h to allow the worms to evacuate any non-adhered bacterial cells from the intestine. Worms were then rinsed twice with M9 Buffer, cooled down 15 min at 4 °C to stop peristalsis, and bleached for 6 min at 4 °C with 100 µL M9 Buffer + 0.2% v/v bleach (Clorox). Worms were then rinsed three times with cold M9 Buffer + 0.1% Tx to remove the bleach.

Manual disruption with a motorized pestle and 96-deep-well plate disruption with silicon carbide grit followed previously described protocols [35]. To guarantee the background media was fully clean, the supernatant in each sample was also collected, serially diluted, and plated onto Nutrient Agar (3 g yeast extract, 5 g peptone, and 15 g of agar [Bacto] in one liter of water). The plates were incubated at room temperature for 2 days to allow distinct colony morphologies to develop, and then the colonies were counted with the aid of a stereo microscope (Leica MZ10 F).

### Co-culture experiments in vitro

Utilizing the same 1% AXN medium, 96-deep-well culture plates, and 150 µl volume per sample used to colonize *C. elegans*, pairs of bacterial species were mixed at a concentration of 10^5^ CFU/ml each. This inoculum concentration is lower than in the worm colonization experiments to allow growth for all bacteria (Fig. S1E). We allowed the bacterial relative abundances to equilibrate with seven growth-dilution cycles, where the bacteria are diluted 100-fold into fresh media each day. Bacterial abundances were quantified by plating onto agar and distinguishing colony morphologies. To lower the pH of the S medium + 1% AXN, NaOH 1M was added while measuring continuously the pH of the media with a microelectrode (N6000BNC, SI Analytics). The media was filtered (Millex-SV 0.2 µm, MerckMillipore) afterwards.

### Data analysis

Pairwise and trio outcomes were categorized as coexistence if the rare species was present at an average abundance of more than 2%. This threshold is just above our usual limit of detection of ~1%, which is inversely proportional to the number of colonies counted. The pair *Pf-Ea* (1.7%–98.3%) was defined as coexisting since we could reassure the presence of *Pf* with more than one biological replicate. Given that we fed the worms with one initial composition, we cannot detect the possibility of bistability, in which the final fractional abundance depends upon the starting ratio of the two species.

The mean relative yield of a species in a co-culture, *RY*_*i*|*j*_, was calculated by bootstrapping (sampling with replacement) simultaneously over the pairwise and monoculture CFU/worm data to obtain vectors *N*_*i|j*_ and *N*_*i*_, respectively, and then calculating $log(RY_{i|j}) \,=\, \left\langle {log((N_{i|j} \,+\, 1)/\left\langle {N_i \,+\, 1} \right\rangle )} \right\rangle$. We utilized logarithmic scales to have comparable calculations regardless of numerator/denominator choice, and we added 1 to avoid ±infinite. The null expectation based on monocultures is obtained by averaging the fractional abundances of all possible combinations of the species’ monoculture information. The standard error of the mean (s.e.m.) is calculated with *n* as the least number of monoculture replicates. These mean and s.e.m. can also be obtained by bootstrapping over the monoculture data. The hierarchy score of the fractional abundances’ matrix was calculated as previously described [42] (Supplementary Information).

For phylogeny reconstruction, sequences of the full 16S rRNA gene were obtained from NCBI. *Sulfolobus solfataricus*, a thermophilic archaea, was used as an outgroup species to root the tree. Clustal X with default parameters was used to align the sequences [43]. PhyML-SMS with default parameters was used to select GTR + G + I as the best model and to infer the tree [44]. The phylogenetic distances were calculated directly from the phylogenetic tree.

## Results

### Monocultures differ significantly in their ability to colonize the *C. elegans* intestine

To investigate community assembly in the gut of *C. elegans*, we fed germ-free synchronized adult worms with different bacterial species, in monoculture or in mixture, over 4 days in a well-mixed rich liquid medium (Methods, Fig. S1A). The majority of worms survived the 4-day period of feeding and colonization, after which we allowed live worms to feed briefly on heat-killed *E. coli* OP50 to remove transient colonizers [35, 45]. We then cleaned the surface of the worms with consecutive washes, and measured the intestinal bacterial densities by grinding batches of worms, plating, and counting colony forming units (CFU, Fig. S1B) with distinct morphologies [46]. The supernatant of each sample was plated to verify that CFU counts came from the worm digestions instead of the background media (Methods). This protocol allowed us to construct and quantify simple microbiotas in *C. elegans*.

We began by feeding *C. elegans* in monoculture to quantify the ability of a range of bacterial species to colonize and grow in the worm intestine. As a starting point, we first utilized an immunocompromised *C. elegans* mutant (AU37) and a set of eleven non-native bacterial species (Fig. 1B), representing the phyla Firmicutes (gram-positive) and Proteobacteria (gram-negative). We found that all bacterial species colonize (i.e., accumulate with or without active growth) the *C. elegans* intestine, with mean population sizes (Figs. 1C, S1C) ranging from 200 CFU per worm in the case of *B. cereus*, up to 20,000 CFU/worm in the case of *S. marcescens*. Our three Firmicutes reach low population sizes in the worm gut and low carrying capacities in the liquid media (Fig. S1E), but the carrying capacities in the liquid media do not explain the variation in monoculture colonization (Fig. S1F, G). These results indicate that different non-native bacterial species have a wide range of abilities to colonize the *C. elegans* intestine in monoculture.

### Composition of two-species microbiotas are influenced by competitive and hierarchical bacterial interspecies interactions

**Fig. 2:**
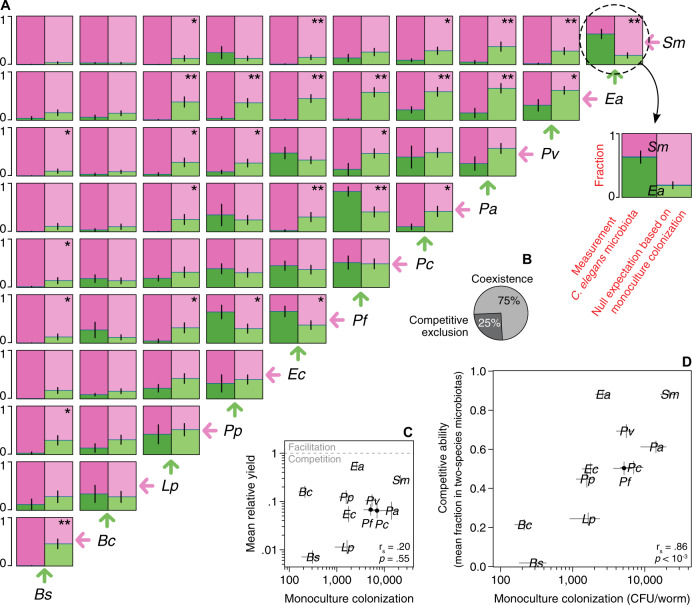
Monoculture colonization of the worm intestine often fails to predict composition of two-species microbiotas. **A** LEFT panels: Fractional abundances of 55 co-culture experiments in *C. elegans* intestine (AU37); error bars are the s.e.m. of 2–8 biological replicates (Fig. S2). Bacterial species are ordered from left to right by their mean fraction across all co-cultures. RIGHT panels: Null expectation for the fractional abundances based on a noninteracting model where each bacterial species reaches its population size in monoculture; error bars are the s.e.m. from bootstrapping over the monoculture data. * and ** represent a statistically significant difference between the two panels at *p* values of 0.05 and 0.01, respectively (Welch’s *T* test). **B** Coexistence of two species is more common than competitive exclusion in the worm intestine. **C** Low yields in two species microbiotas—relative to monocultures—are indicative of competitive interactions (Fig. S2); error bars on *X*-axis are the s.e.m. and on *Y*-axis the s.e.m. from bootstrapping over monoculture and pairwise data simultaneously. **D** Competitive ability, defined as the mean fractional abundance in co-culture experiments, relates to monoculture population size, but there are significant deviations; error bars on *Y*-axis are the propagated error from the s.e.m. of the co-culture experiments.

To assess the compositional trends of the *C. elegans* microbiota, we constructed the simplest intestinal communities in this worm by feeding it with all possible two-species mixtures from the same eleven non-native bacteria as before (55 pairs, Figs. 2A, S2A). We fed worms with both bacteria present at similar concentrations (~10^7^ CFU/mL, Methods) to normalize the rate of ingestion. We found that a majority (41 out of 55, ~75%) of pairs displayed coexistence, with both species present above the detection limit of 2%, whereas the remainder (14 out of 55, ~25%) led to competitive exclusion of a species (Figs. 2B, S2B). These results show that bacteria with no prior conditioning for the *C. elegans* gut commonly reach coexistence in two-species microbiotas.

The interactions between bacterial species in a microbiota can be classified as positive, negative, or neutral based on the *yields* of the bacteria *relative* to their monoculture population sizes. To classify the interactions in our two-species microbiotas, we calculated the relative yield of species “i” with species “j”, *RY*_*i|j*_, as its population size in co-culture, *N*_*i|j*_, divided by its population size in monoculture, *N*_*i*_ (*RY*_*i|j*_ = *N*_*i|j*_/*N*_*i*_, see Methods for detailed implementation). We found that most species cannot reach their monoculture population size in co-culture experiments, *RY* < 1 (Figs. 2C, S2D, E), suggesting that interactions between species are largely competitive [14, 47, 48]. From our 110 *RY* measurements, only *Ea* co-cultured with *Pa* reached a *RY* significantly greater than 1. Furthermore, most co-cultures reach lower community sizes than the higher population size of the monocultures (Fig. S2D), indicating that the observed low relative yields are not simply due to competition for fixed space within the worm gut. Interestingly, the mean relative yield of each species does not correlate with its monoculture colonization (Spearman correlation *r*_s_ = 0.20, *p* = 0.55, Fig. 2C), indicating that a large population size does not protect a bacterial species from being harmed by competition in co-culture experiments.

To explore the influence of monoculture colonization ability, which does not depend on interspecies interactions, on the assembly of the *C. elegans* microbiota, we further compared the monoculture population sizes and the two-species microbiotas. We first simplified the worm pairwise outcomes into a summary-metric by calculating the mean fractional abundance of each species in all two-species microbiotas, $\left\langle {F_i} \right\rangle _{{\mathrm{v}}j} \,=\, \langle {\frac{{N_{i|j}}}{{( {N_{i|j} \,+\, N_{j|i}} )}}} \rangle _{{\mathrm{v}}j}$. We found that this competitive ability score correlates to the population size reached in monoculture colonization (Fig. 2D, *r*_s_ = 0.86, *p* < 10^–3^). This positive relationship indicates that a bacterial species persists in two-species microbiotas due to similar properties to those favoring its monoculture colonization of the gut. This, together with the previous result, indicates that the uneven harm caused by competition does not dramatically alter the mean fractional abundance expected from monocultures.

Despite this correlation between monoculture colonization and competitive ability, some species in co-culture performed differently than would be expected based simply on population sizes. For example, *Ea* tied with *Sm* for being the strongest competitor despite being only the sixth highest colonizer in monoculture (Fig. 2D). We therefore sought to determine to what extent interactions between microbes are important to predict the pairwise outcomes in the host. We calculated a null expectation for the two-species microbiotas assuming that each species is able to reach the carrying capacity that was measured in monoculture colonization (Fig. 2A, right panels). By comparing this null expectation with the experimentally measured fractions obtained from pairwise colonization (Fig. 2A, left panels), we are able to identify the cases in which interspecies interactions play a dominant role in determining the composition of the gut microbiota [47, 49]. In 28 out of 55 cases this deviation is large enough to reject the null model (*p* < 0.05, Fig. 2A), many more than the 2.75 cases expected by chance at this significance level (16 cases with *p*_FDR_ < 0.05). These results indicate that a null expectation, where each species’ abundance in pairwise colonization is determined by its monoculture fitness to the worm gut environment, rather than by interactions between bacteria, fails to predict a significant number of two-species microbiotas.

To further characterize the structure of the competition network, we quantified its degree of hierarchy [42], which estimates how frequently a highly ranked competitor will dominate a lower-rank adversary. The hierarchy score of this network, 0.82, is significantly larger than the hierarchy score found in random matrices with the same distribution of fractional abundances (*p* < 10^–5^, Supplementary Information), suggesting that there is an approximate ordering of the competitive abilities of these bacterial species in the worm gut. Consistent with this ordering, we do not observe any cases of intransitive competition, in which the pairwise interactions of three bacterial species would be analogous to the rock–paper–scissors game and no absolute winner would exist [50, 51]. This intransitivity has been proposed as a major mechanism inducing coexistence in natural populations [52–56], but we do not observe it in the pairwise interactions of any of our 165 hypothetical trios. With a more relaxed definition of intransitivity, in which a species wins a competition by being more abundant than the competitor instead of needing to fully exclude it, we find two candidate trios with a rock–paper–scissors-like structure: *Ec-Pf-Pa* and *Pp-Pf-Pa* (although the dominance of some competitors is not statistically significant).

Collectively, we find that the monoculture colonization ability of a bacteria correlates with its mean abundance in two-species microbiotas. Additionally, the interspecies interactions, which are mostly competitive and hierarchical, alter the composition of at least half of the individual two-species microbiotas (Fig. 2).

### Three-species microbiotas are predicted by pairwise outcomes, not by monocultures

**Fig. 3:**
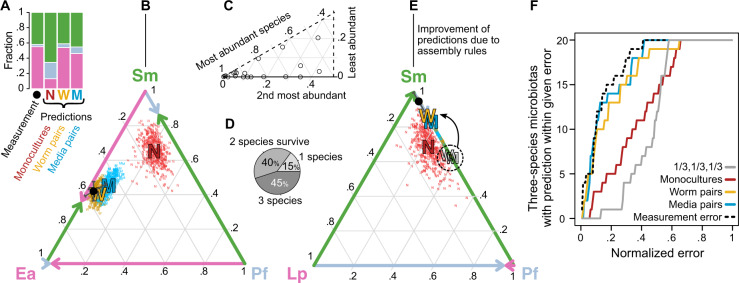
Fractional abundances in three-species microbiotas are well predicted by pairwise outcomes. **A** Outcome of trio *Ea-Pf-Sm* in *C. elegans* (AU37) intestine, together with predictions based on monoculture population sizes, two-species microbiotas, or pairwise outcomes in vitro liquid media (normalized arithmetic mean). **B** Simplex representation of trio outcome and predictions in (**A**), with the edges of the triangle depicting the two-species microbiotas in *C. elegans*. The error bars on measurement are the s.e.m. of four biological replicates, and the clouds of points around predictions are 400 bootstrap replicates (“N”s sampling the monoculture data, and “W”s and “M”s sampling the pairwise data in worm and media, respectively). **C** Twenty trio outcomes represented in one sixth of a simplex. **D** 3, 8, and 9 out of the 20 trios show full competitive exclusion, two- and three-species coexistence, respectively. **E** Assembly rules help the quantitative prediction of the trio outcomes based on pairwise outcomes when one of the pairs is competitive exclusion. **F** Cumulative distribution of error of predictions. Error calculated as the linear distance between prediction and measurement in the simplex. The distances are normalized by the maximal distance, √2. The dashed line is the mean distance between the measured mean and the four biological replicates of each trio, and serves as a lower bound for the error of the predictions.

In the two-species microbiotas, we observed frequent coexistence of non-native bacteria in the *C. elegans* gut, but it remains to be tested if coexistence is also the norm in gut communities initialized with a larger number of species. We therefore constructed 20 three-species microbiotas in *C. elegans* to extend our analysis. From our eleven non-native bacterial species, we selected a set of six (*Bc, Lp, Pf, Pv, Ea*, and *Sm*) that span the range of competitive abilities observed, and we fed them in all possible trios to *C. elegans* (Fig. S3A). In the trio *E. aerogenes-P. fluorescens-S. marcescens*, for example, we observed coexistence of the three species, in which *Ea* is the majority, *Sm* is close second, and *Pf* is a minority (55 ± 7%, 42 ± 5%, 3 ± 3%, respectively; mean ± s.e.m.). These fractional abundances can be represented as a stack of bars (Fig. 3A) or as a point in a simplex (Fig. 3B), where the point moves closer to a vertex when that given species increases in abundance. By plotting the measurements of all 20 trios into one simplex (Fig. 3C), we observe that most of the cases have one species as highly abundant, yet full exclusion is rare and only accounts for 3 out of the 20 trios tested (Fig. 3D). Our trio feeding experiments therefore display a range of different outcomes, with frequent coexistence of the three species leading to multispecies gut microbiotas.

To test if monoculture colonization contains the information necessary to estimate the assembly of three-species microbiotas, we made quantitative predictions of the trio outcomes based on monocultures. We extended the null expectation described earlier by assuming that all species will reach their population sizes in monoculture colonization (“N” Figs. 3, S3). This null expectation achieves poor results at predicting trio outcomes, for its mean error of 35.7% is just somewhat better than the 43.8% mean error of an uninformed “^1^/_3_, ^1^/_3_, ^1^/_3_” prediction (Fig. 3F). Hence, monocultures are inadequate at predicting three-species microbiotas, highlighting that as community diversity increases, the properties favoring monoculture colonization of the gut are less important than the interspecies interactions for determining community composition.

To determine the role of bacterial pairwise interactions on the assembly of the *C. elegans* microbiota, we made predictions of all three-species microbiotas based on two-species microbiotas. We first calculated a simple linear prediction for each trio by taking the arithmetic mean of each species’ fraction in the co-culture experiments against the other two species (a normalization factor of 2/3 is needed for the fractions of the three species to add to one). This *normalized arithmetic mean* prediction, applied over the two-species microbiotas in the worm (“W” Figs. 3A, B, S3A), quantitatively predicts some trios with high accuracy, and exhibits a mean error of 26% (Fig. S3C). However, this prediction is prone to error (hollow “W” Fig. 3E) when one of the two-species microbiotas is competitive exclusion. A recently proposed assembly rule [40] is capable of adjusting these cases by simply removing a bacterial species from the trio prediction when it cannot survive both constituent co-culture experiments (solid “W” Fig. 3E). After the application of this assembly rule, the mean error of the predictions based on two-species microbiotas, 18.7%, comes close to the expected biological noise in three-species microbiotas, 13.3% (Fig. 3F). The fact that two-species microbiotas can properly predict three-species microbiotas indicates that interactions between pairs of bacterial species are an important force in determining microbiota assembly and suggests that indirect higher-order interactions are uncommon or weak.

Our results diverge from three recent findings, where higher-order interactions played a role in the zebrafish microbiota assembly [14], changed an in vitro community amylolytic function [57], and prevented invasion of an algae-bacteria-ciliate community [58]. The discrepancy may be due to the interaction-estimation approach used there and the simpler rules-based approach [40] used here, and/or due to biological differences in the model systems. However, consistent with what we report here, recent experiments have found that two-species microbiotas are able to predict the fitness traits of flies with multispecies microbiotas [17].

Next, we asked whether it would be possible to make predictions of the 20 three-species microbiotas based on the pairwise outcomes from a different environment, such as the in vitro liquid media used as feeding substrate. Thus, we performed all possible pairwise co-culture experiments in liquid media without worms and measured the equilibrated bacterial fractions after seven cycles of 100-fold daily dilution (Methods, Fig. S4, further explored in section below). After applying the assembly rules, the mean error of the predictions based on media pairwise outcomes, 15.7%, also comes close to the expected biological noise of the three-species microbiotas (Figs. 3F, S3A). Since the equilibrium fractional abundances in the liquid media are dependent on the dilution regime [59], our results highlight that our chosen daily dilution of 100-fold can resemble the pairwise outcomes in the worm gut. These results show that three-species microbiotas in *C. elegans* with non-native bacteria can still be predicted with the pairwise outcomes measured in a different environment, suggesting that the environmental filtering of the worm intestine is not the main determinant of community assembly.

### Bacterial relative abundance in *C. elegans* microbiota is dependent on phylogeny rather than isolation origin

Thus far we have explored the assembly of the *C. elegans* microbiota with non-native bacteria (Fig. 1B). These laboratory species were not isolated from any worm gut, and they hadn’t been conditioned to grow in the *C. elegans* gut environment before the beginning of each of our experiments. Using these non-native bacteria helped us reduce the complexity associated with microbiota assembly by setting aside the selection force that *C. elegans* could have exerted on its microbiota during evolutionary timescales. Within our non-native species we observed strong and weak colonizers, strong and weak competitors, and an imperfect relation between these two metrics because of interspecies interactions. It remains to be seen if these observations hold true with bacteria native to the *C. elegans* gut.

**Fig. 4:**
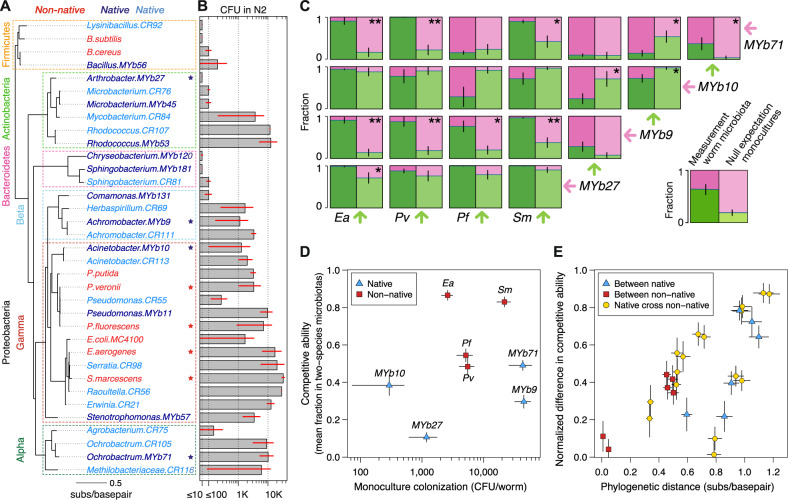
Experimental colonization of *C. elegans* by a wide range of native and non-native bacteria reveals that phylogeny rather than isolation origin determines abundance in the gut microbiota. **A** Phylogenetic classification of previously shown laboratory species (non-native) and bacterial strains isolated from *C. elegans* intestines (native; dark and light blue from MYb and CR collections, respectively; Methods). Phylogenetic tree built with maximum likelihood estimate utilizing alignment of full-length 16S gene sequences. The phylogenetic tree is sorted at each internal node to have the higher monoculture colonizers at the bottom. High level phylogenetic classification is given on the left side of the tree for ease of interpretation. Stars indicate bacteria used in follow-up two-species microbiotas. **B** Bacterial population sizes in monoculture colonization of wild-type *C. elegans* (N2); error bars are s.e.m. of two to three replicates. **C** Left panels: Fractional abundances in two-species microbiotas with native and non-native bacteria in *C. elegans* intestine (AU37). Right panels: Null expectation for the fractional abundances based on monoculture population sizes. “*” and “**” represent a statistically significant difference between measurement and null expectation at *p* values of 0.05 and 0.01, respectively (Welch’s *T* test). **D** Although two native strains can reach substantial colonization of the worm intestine in monoculture, these strains reach low fractional abundances in two-species microbiotas. **E** Differences in competitive ability correlate with phylogenetic distances regardless of the isolation origin of the bacteria. Phylogenetic distances are the horizontal distances in the phylogenetic tree. Differences in competitive ability are normalized by the maximum competitive ability of the pair (i.e., competitive abilities 0.8 and 0.4 are as different as 0.2 and 0.1).

Previous studies have assessed the natural *C. elegans* microbiota [18] by isolating and cultivating nematodes in decaying organic matter, and have isolated native bacterial strains by grinding the naturally colonized worms [30–32, 45]. We isolated new bacterial strains from worms with a similar protocol (CR collection, Methods) and also utilized the MYb collection from Dirksen et al. [32, 60] which contains some of the bacteria persistently found in the *C. elegans* gut. From these two collections, we selected a phylogenetically diverse set of bacterial isolates, spanning four different phyla, to study the colonization patterns of native bacteria (Fig. 4A, 12 MYb, 15 CR).

To characterize the monoculture colonization ability of these 27 native bacterial isolates, we fed them in monoculture to wild-type *C. elegans* (N2, from where they were isolated) along with eight of the previous non-native bacteria (Fig. 4B). We found that the native and non-native bacteria colonize in a similar fashion. Regardless of the origin of the isolate, the Firmicutes colonized poorly and the Gammaproteobacteria often colonized well, resulting in monoculture population sizes that in some cases exceeded 10,000 CFU/worm. By comparing 16S phylogenetic similarity between each pair of bacteria (Fig. S5A) against their fold-difference in monoculture colonization (Fig. S5B), we observed that similar bacteria colonize similarly, and as phylogenetic distance increases, the difference in colonization ability tends to increase as well (*r*_s_ = 0.39, *p* = 0.003, Mantel test). This positive correlation is true regardless of the native/non-native dichotomy, and can be observed within the genera *Bacillus*, *Microbacterium*, *Sphingobacterium, Serratia, Ochrobactrum*, etc. (Fig. 4A, B). Overall, these results show that monoculture colonization ability is similar between native and non-native bacterial strains, and suggest that this metric depends on evolutionary history rather than isolation origin.

The possible host–microbe adaptation of the native bacterial isolates to the worm gut could have selected for stronger bacterial competitors, besides the selection for health-promoting bacteria that has been previously reported [61]. To investigate how the competitive ability of native bacteria compares to that of non-native bacteria, we performed further feeding experiments in *C. elegans* with all monocultures and pairwise combinations of four native isolates and four non-native species (stars in Fig. 4A; Natives selected to cover the range of monoculture colonization, expand our phylogenetic diversity, and display distinct colony morphologies: *Achromobacter MYb9*, *Acinetobacter MYb10*, *Arthrobacter MYb27*, and *Ochrobactrum MYb71*; Non-native: *Ea*, *Pv*, *Pf*, and *Sm*). The measured fractional abundances in immunocompromised AU37 worms showed that the composition of two-species microbiotas once again often deviates from a null expectation based on monoculture colonization (Fig. 4C, 4/6 cases for between native pairs, and 8/16 for native cross non-native pairs with *p* < 0.05). In this new set of experiments involving native isolates, more interactions appear to be positive (in the form of parasitisms), with *Ea*, *Sm*, and *MYb10* being facilitated the most (Fig. S5C, D). Moreover, we found that non-natives reach higher mean fractional abundances than native bacteria in the two-species microbiotas (Fig. 4D). *Ochrobactrum MYb71* and *Achromobacter MYb9* had the largest monoculture population sizes yet low fractional abundances in two-species microbiotas, indicating a low competitive ability, while *Acinetobacter* *MYb10* showed the opposite characteristics. Further comparison of native and non-native bacteria is warranted, but our two-species microbiotas indicate that native bacteria also interact in the digestive tract of *C. elegans* to structure the microbiota composition (Fig. 4C). These native isolates lack a clear competitive advantage over non-native bacteria, particularly when co-cultured with strong competitors (*Ea, Sm;* Fig. 4D).

To test if phylogenetic differences are responsible for the observed differences in competitive ability, we compared these two metrics using our set of native and non-native bacteria (Fig. 4E). We observed that the competitive abilities of a pair of bacteria differ more as the bacteria diverge phylogenetically (16S gene), regardless of the isolation origin. Controlling for phylogenetic distance, a bacterial strain native to the *C. elegans* gut appears equally different from another native strain or a non-native strain (Fig. 4E). Our data therefore suggest that the composition of two-species microbiotas in *C. elegans*, as well as the more basic monoculture colonization, is determined more by a species’ phylogenetic classification than by whether the species was isolated from the worm microbiota.

### Environmental filtering by *C. elegans* gut can alter pairwise outcomes

**Fig. 5:**
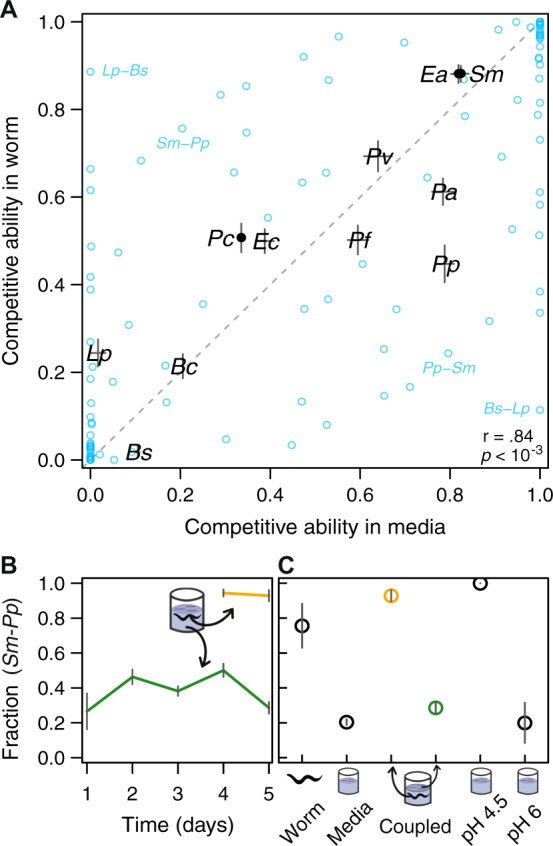
Bacterial interspecies interactions are similar between the in vitro and in vivo environments, with some differences caused by the acidity of the worm gut. **A** Black points are the mean fractional abundance in co-culture experiments in *C. elegans* intestine and liquid media (1% AXN); error bars are the propagated error from the s.e.m. of the underlying co-culture experiments. Blue points are the outcomes of individual co-culture experiments in worms and media. **B** *S. marcescens* and *P. putida* reach different fractional abundances in vivo worm gut and in vitro liquid media on a coupled experiment, where worms and liquid media from the same test tube are tested. **C** An acidic version of the media resembling the average pH of the worm intestine (4.5) shifts back the pairwise outcome to a worm-like state; error bars are the s.e.m. of at least four replicates.

Previous detection of bacterial genera enriched in the *C. elegans* intestine compared to the substrate where the worms were grown, such as *Ochrobactrum*, has suggested an important role for environmental filtering by the *C. elegans* gut during microbiota assembly [62]. Nevertheless, our results, showing that three-species microbiotas are well predicted by in vitro pairwise outcomes (“M” Fig. 3), suggests that environmental filtering by the worm intestine is not the main determinant of community assembly for this set of bacteria. To further characterize the effect of the *C. elegans* gut environment on bacterial community assembly, we directly compared the outcomes of 55 co-culture experiments between in vivo worm gut and in vitro liquid media (Figs. 5A, S4A, Methods). We found that competitive ability (Fig. 5A, *r* = 0.84, *p* = 0.0005) and mean relative yield (Fig. S4B, C, *r* = 0.81, *p* = 0.0018) in the worm gut and liquid media are correlated, which suggests that the environmental filtering that *C. elegans* provides is not strong enough to alter the hierarchical ordering of these eleven bacterial strains. Although competitive ability is similar between the worm gut and in vitro liquid media, we found that 19 out of 55 bacterial pairs displayed a significantly different outcome in these two environments (*p* < 0.05, Fig. S4A, D). From these 19 bacterial pairs, we found nine displaying coexistence in the worm yet competitive exclusion in the liquid media, while zero pairs displayed the opposite trend, indicating that the worm intestine allows for more coexistence. Our data therefore indicates that the *C. elegans* intestine doesn’t alter the competitive hierarchy of its bacterial colonizers, but is capable of altering specific pairwise outcomes.

In order to find the features that differentiate the worm gut and the liquid media, we set off to investigate more closely one bacterial pair, *S. marcescens-P. putida*, in these two environments. *Sm* is a minority of the in vitro community (20 ± 2%) yet is a majority in the worm gut (76 ± 12%, Fig. 5A). We first fed a population of worms with a mixture of *Sm-Pp*, but instead of restoring the liquid feeding substrate every day to maintain equal proportions of both species, we allowed the bacteria to be carried over with the dilution step. In this coupled co-culture experiment, where strong migration occurs between worms and media, we still observed different compositional states in the different environments (Fig. 5B). This result shows that the environmental filtering imposed by the worm intestine can be strong enough to keep an internal bacterial community different from its surrounding environment.

Given the importance of pH to microbial growth and competition [1, 63, 64], we tested whether the low pH of the worm gut could cause the persistent difference across environments for the *Sm-Pp* bacterial pair. We therefore repeated the co-culture experiment in liquid media at its normal pH 6, and at lower pH 4.5, where the latter approximates the conditions within the nematode intestine [65, 66]. For this pair of species, lowering the pH of the media was sufficient to alter the pairwise outcome in vitro, resulting in a community very similar to that observed in the worm intestine (Fig. 5C). Similar results were observed in the pair *Lp-Bs* (Fig. S4E, F). We conclude that *C. elegans* and its feeding substrate can reach different stable states, and the acidic pH of the worm gut can be an important component of the environmental filtering by this host during community assembly.

### Innate immunity of *C. elegans* reduces bacterial loads, but has little effect on microbiota composition

**Fig. 6:**
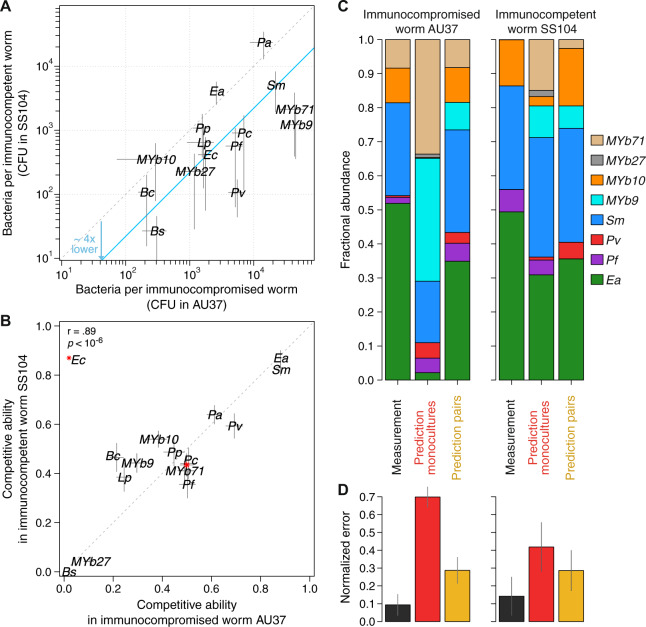
Innate immunity of *C. elegans* via the p38 MAPK pathway reduces bacterial population sizes, but has little influence on the composition of the two- and eight-species microbiotas. **A** Immune system of *C. elegans* reduces bacterial monoculture population sizes unevenly for different bacteria. Immunocompromised *C. elegans* (AU37) has larger bacterial population sizes in its intestine than immunocompetent *C. elegans* (GLP4). **B** The mean fractional abundances in co-cultures are similar between the two worm strains with different immune activity. **C** Composition of an intestinal microbiota in immunocompromised *C. elegans* AU37 and immunocompetent SS104, together with predictions based on monoculture colonization and pairwise outcomes in the same worm strains. Three or more batches of ~20 worms digested for each measurement. **D** Errors are the L1 norm (Manhattan distance) between measurement replicates and predictions. The variability across different batches of digested worms generates a measurement error of 9.3% and 14.2% for AU37 and SS104, respectively. The errors are normalized by 2, the maximum error. Confidence intervals of the prediction errors were calculated by bootstrapping over the corresponding data.

To further our understanding of the effect that *C. elegans* has in the assembly of its microbiota via its immune system, we set off to compare equivalent one- and two-species microbiotas in two *C. elegans* strains with different immunity levels (Fig. 6). The *C. elegans* strain AU37 is susceptible to high bacterial colonization due to its deleted *sek-1* gene, which encodes for a kinase part of a signaling cascade homologous to the human p38 MAPK (mitogen-activated protein kinase) pathway [67, 68]. The *C. elegans* p38 MAPK pathway activates the production of immune effector molecules, such as lysozymes *lys-2* and *lys-8* [69]. The *C. elegans* strain SS104 has the same *glp-4* mutation as AU37 that leads to sterility at room temperature, allowing us to work with same-size, synchronized worms. While the strain SS104 is not immunologically wild-type—this mutant shows up-regulation of the DAF-2/IGF pathway, presumably as a by-product of its reproductive sterility [45, 70, 71]—it is the immunocompetent counterpart of AU37 in that it has its wild-type *sek-1* gene and therefore intact signaling through its p38 MAPK pathway. Comparison of the gut microbiota communities in the worm strains SS104 and AU37 therefore allows us to directly study the role of the worms’ p38 innate immunity pathway in structuring its gut microbiota.

We first explored the monoculture colonization of the immunocompetent *C. elegans* SS104 by feeding it with the 11 non-native and four native bacterial strains shown before. We found that all bacterial species colonize the SS104 intestine, with mean population sizes ranging from 30 CFU/worm in the case of *Bs*, to more than 10,000 CFU/worm for *Pa* (Figs. 6A, S1C). On average, the bacterial population sizes in the p38-immunocompetent *C. elegans* were ~4 times lower than in the immunocompromised AU37 strain, but this reduction in bacterial load was uneven across species. For example, *Ea*, *Pa*, and *Acinetobacter MYb10* seemed to reach similar carrying capacities regardless of p38 MAPK pathway activity, while the population sizes of *Bs*, *Pv*, and *Achromobacter MYb9* were reduced by more than 90% when this pathway was active. These results show that the innate immune system of *C. elegans* via its p38 MAPK pathway reduces population sizes of most bacterial strains, but some colonizers are less affected than others.

In order to test the effect of the *C. elegans* immune system via p38 MAPK on the composition of two-species microbiotas, we fed immunocompetent SS104 with all bacterial pairwise combinations previously studied in immunocompromised AU37 (55 pairs from 11 non-native bacteria, 6 pairs from 4 native bacteria, and 16 pairs from 4 native vs. 4 non-native bacteria; 77 pairs in total; Fig. S6A, B). We found a strong correlation between the mean fractional abundances in the two worm strains (Fig. 6B), with *Ea* and *Sm* as the best competitors, and *Bs* and *Arthrobacter MYb27* as the worst competitors in both worm mutants. We also observe a majority of the two-species microbiomes displaying similar abundances in the two worm strains (Fig. S6A, C). These results show that the *C. elegans* immunity via its p38 MAPK pathway has little effect on the composition of two-species microbiotas.

Moving into more complex microbiotas, we built, measured, and compared an eight-species microbiota in our two worm strains AU37 and SS104. We utilized the same set of native and non-native strains explored in pairwise combinations before (Fig. 4D). After 4 days of feeding, we measured the bacterial abundances in the ensuing microbiotas (Fig. 6C). The three most abundant bacterial species were the same in both worm strains: *Ea*, *Sm*, and *Acinetobacter MYb10*. In accordance with the results of two-species microbiotas, the two *C. elegans* strains with different immunity levels displayed very similar average microbiota compositions (L1 norm distance = 1.4%, lower than the L1 norm measurement error for AU37 = 9.3% and SS104 = 14.2%, Fig. 6D). These results show that the *C. elegans* immunity via its p38 MAPK pathway also has little effect in the composition of microbiotas with higher richness.

To test how predictable this eight-species microbiota is, we made predictions of it based on monocultures and pairwise outcomes (Fig. 6C, D). As before, we first calculated a null expectation assuming that each species is able to reach the carrying capacity that was measured in monoculture colonization. This prediction based solely on monocultures reached a mean error of 70 ± 5% in immunocompromised AU37, and 42 ± 13% in the immunocompetent SS104. These high errors show once again that information on monoculture colonization is insufficient to predict the assembly of microbiotas, which highlights the importance of interspecies interactions. We then utilized the two-species microbiotas to predict the eight-species microbiota in the two *C. elegans* strains (Fig. 6C, D). Only the bacterial strains that were not competitively excluded in co-cultures should survive in the larger microbiota (assembly rules), and then we predicted the abundance of each survivor as its mean fractional abundance in co-culture experiments against the other survivors (arithmetic mean prediction). This prediction based on pairs reached an error of 28 ± 7% in immunocompromised AU37, and 28 ± 11% in immunocompetent GLP4, smaller errors than the predictions based on monocultures. Collectively, these data suggest that pairwise interactions are useful estimators of larger microbiotas.

## Discussion

Here we characterized the bacterial colonization of the *C. elegans* intestine by native and non-native strains. We observed across three different environments (p38-immunocompetent *C. elegans* SS104, p38-immunocompromised AU37, and in vitro liquid media) a similar ordering of all the bacterial strains based on their competitive ability, indicating that the environmental filtering by the *C. elegans* intestine and by its immune system modify only a subset of the bacterial communities tested. Overall, our results show that bacterial interspecies interactions strongly influence the composition of 2-, 3-, and 8-species microbiotas, while monoculture colonization, isolation origin of bacteria, environmental filtering by the worm intestine, and immune system of the worm play secondary roles in the assembly process.

The worm gut environment is capable of enriching for certain species from the surrounding environment [32], and bacteria can evolve higher competitive abilities after in vivo passages within *C. elegans* [61, 72] (similar results in zebrafish [73] and tomato plants [74]), but our results suggest that strains isolated from the gut of natural *C. elegans* are not adapted to form bigger populations or to be better competitors than strains isolated elsewhere. A possibility for this discrepancy is that the adaptation of the microbes is rather specific to the exact hosts from where they were isolated, as it has been suggested for the bee microbiota [24]. Other possibilities are that host–microbe adaptation hasn’t occurred yet with the MYb bacteria that we probe, or that the adaptation that occurred selected for a different trait instead of stronger colonizers, such as healthy microbes for *C. elegans* [7, 45]. Further experiments are warranted, but our results showing non-native *E. aerogenes* and *S. marcescens* as the fittest bacteria for the *C. elegans* intestine, which align with previous results showing Enterobacteriaceae and Pseudomonadaceae as the most abundant bacterial clades in the natural *C. elegans* microbiota [30–32, 38], suggest that Gammaproteobacteria are intrinsically good at colonizing the worm intestine.

The use of non-native bacteria in the prediction of three-species microbiotas leaves open the question of whether communities with native species can be predicted with in vitro outcomes. Recent work has indicated that native bacteria are potentially functionally important for *C. elegans* [62] and induce specific transcriptional responses in this host [75], providing grounds on which natural selection for host association could occur. It is plausible that greater divergence between in vitro and in vivo community assembly would be seen in a co-evolved community of microbes. Future work comparing bacteria native to *C. elegans* [60] and non-native bacteria will be useful to elucidate the possible host–microbe adaptation occurring in hosts with large flexible microbiotas [10, 18].

We found that most pairwise interactions among non-native species are competitive, but some facilitative interactions appeared in the two-species microbiotas with native species. Further studies should test if native species are indeed more prone to facilitate each other instead of competing. Importantly, the ratio of positive and negative interactions depends on the nutrients in the environment [62, 76], with more nutrients allowing for more bacteria to grow in monoculture, which then leads to more competition [77]. Due to the bacterivore diet of *C. elegans*, a complex mixture of nutrients is perhaps the norm in the *C. elegans* gut, so a rich medium like the one we used is perhaps a good starting point to investigate the *C. elegans* microbiota. Interestingly, we do not observe any cases of strictly non-transitive pairwise interactions (rock–paper–scissors) questioning once more [42] the practical significance of this mechanism at stimulating coexistence and diversity in multispecies communities [56].

The low pH of the gut environment is thought to be a critical factor in host–microbe interactions, and recent work has explicitly demonstrated the importance of pH in modulating the interactions between microbes and determining the structure of synthetic and natural communities [64, 78]. Consistent with these results, we observed that reducing the pH of a liquid medium to simulate the host intestine could alter the outcomes of competition between species and in some cases substantially reduce the difference between in vitro media and in vivo gut.

The antimicrobial defenses activated via p38 MAPK in *C. elegans* SS104, which include lipases and saposin-like proteins, did not substantially shift the microbial composition of two- and eight-species microbiotas in our experiments, which comes in accordance with previous findings [38]. Other signaling pathways in the worm, such as the TGF-ß homologue [38] and insulin-like signaling pathways [37], may be more important in determining microbiota composition, directly and/or by the combined action of multiple pathways.

The work presented here focuses on population averages rather than the composition of bacterial communities within individual worms. Recent results from our group have demonstrated that variation between individuals can be informative––when the feeding densities are lower than the ones used here, there can be an extreme bottleneck during colonization of the worm gut that leads to marked heterogeneity across worms [35]. Similar results have recently been found in *Drosophila* [79], indicating that stochastic effects during colonization may be important in a wide range of host species. At the level of worm populations’ averages, the competitive exclusion observed could be the result of resource competition, including competition for the limited space available within the *C. elegans* gut, or there could be more explicit forms of antagonism such as toxin production [48]. In cases of coexistence, which were the majority in our experiments, spatial partitioning within the host could play an important role, such as was recently found for monoculture colonization of the zebrafish gut [80, 81]. It will be important for future studies to determine the role of stochasticity, priority effects, and spatial dynamics during assembly of multispecies communities within *C. elegans*.

In this study, we have fed worms with defined combinations of bacterial species to elucidate the role of interspecies interactions in the assembly of host-associated microbial communities. These results add to our understanding of how interactions between pairs of bacterial species shape more complex bacterial communities. Our results show that experimental bottom-up microbial ecology is a tool for understanding the dynamics of bacterial gut communities in a simple model organism, providing insight into the forces that shape and control the structure of microbiotas.

## Supplementary information

Supplementary Figures

## Data Availability

All tables of data and code for data analysis will be available upon publication at 10.17632/c5m94tth9n.2.
