# Supplementary material for: Interspecies bacterial competition regulates community assembly in the C. elegans intestine
Source: ISME J. 2021 Feb 15;15(7):2131–45. doi: 10.1038/s41396-021-00910-4 (PMC8245486; doi:10.1038/s41396-021-00910-4)
Supplement: Supplementary file 1 — Supplementary Figures [file 41396_2021_910_MOESM1_ESM.pdf]

Interspecies bacterial competition regulates community assembly in the *C. elegans* intestine

Supplementary Figures

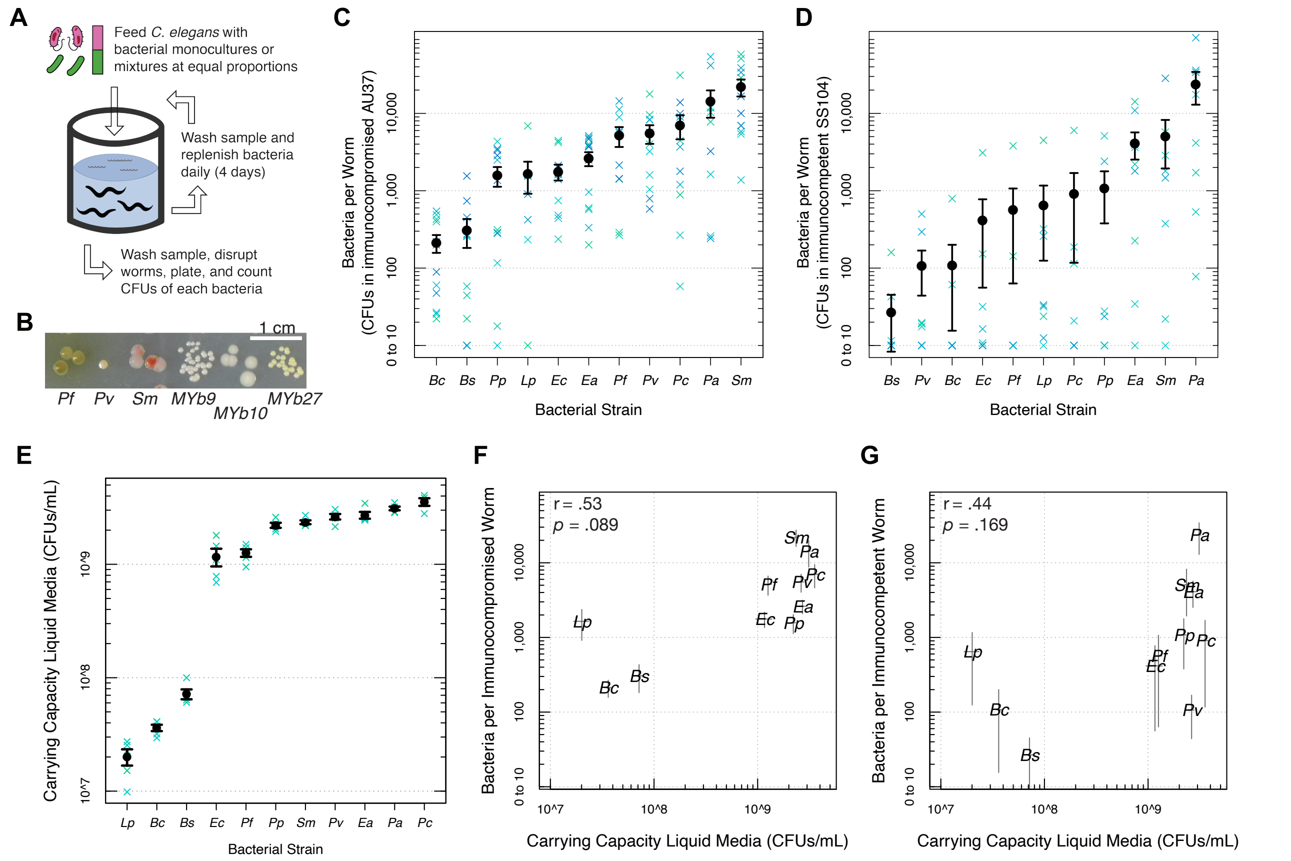

Figure S1. Monoculture colonization of *C. elegans* intestine is variable. (A) To construct and measure simple microbiotas in *C. elegans,* a defined number of bacterial species are fed in liquid culture to a same-age adult population of worms raised on *E. coli* OP50 and sterilized with antibiotics. The liquid feeding substrate is restored every day to maintain equal bacterial concentrations during the four days of colonization. After four days of feeding, worms are mechanically disrupted in batches of ~20, plated onto nutrient agar in triplicates, and the added counts of colony forming units (CFUs) are used to determine bacterial population sizes in the worm gut. **(B)** Photograph of bacterial CFUs of six selected species used in this article, plated onto Nutrient Agar and grown at 25ºC for 2 days (*P. fluorescens, P. veronii, S. marcescens, Achromobacter MYb9, Acinetobacter MYb10, Arthrobacter MYb27*). **(C-D)** Different bacterial species reach widely different average population sizes (black points) during monoculture colonization of the *C. elegans* intestine. Each species also reaches widely different population sizes across biological replicates. Each blue cross is a different mechanical disruption of a batch of ~20 worms, and error bars are the standard error of the mean (s.e.m.). **(E)** Bacterial carrying capacities in the liquid media used as feeding substrate (1%AXN, Methods) were measured by plating the saturated batch cultures (24hrs at 25ºC, OD600 in stationary phase) onto Nutrient Agar and counting CFUs afterwards. The blue crosses show the five biological replicates per species, and the error bars are the s.e.m. **(F-G)**

Comparison of the carrying capacities in the liquid media and the population sizes in the intestine of *C. elegans* AU37 **(F)** and SS104 **(G)**. The three Firmicutes (*Lp*, *Bs*, *Bc*) reach low population sizes in the worm gut and low carrying capacities in the liquid media, but the carrying capacities in the liquid medium don’t explain the variation in monoculture colonization (Pearson correlation coefficients and p-values in the upper left corners). Bars are the s.e.m.

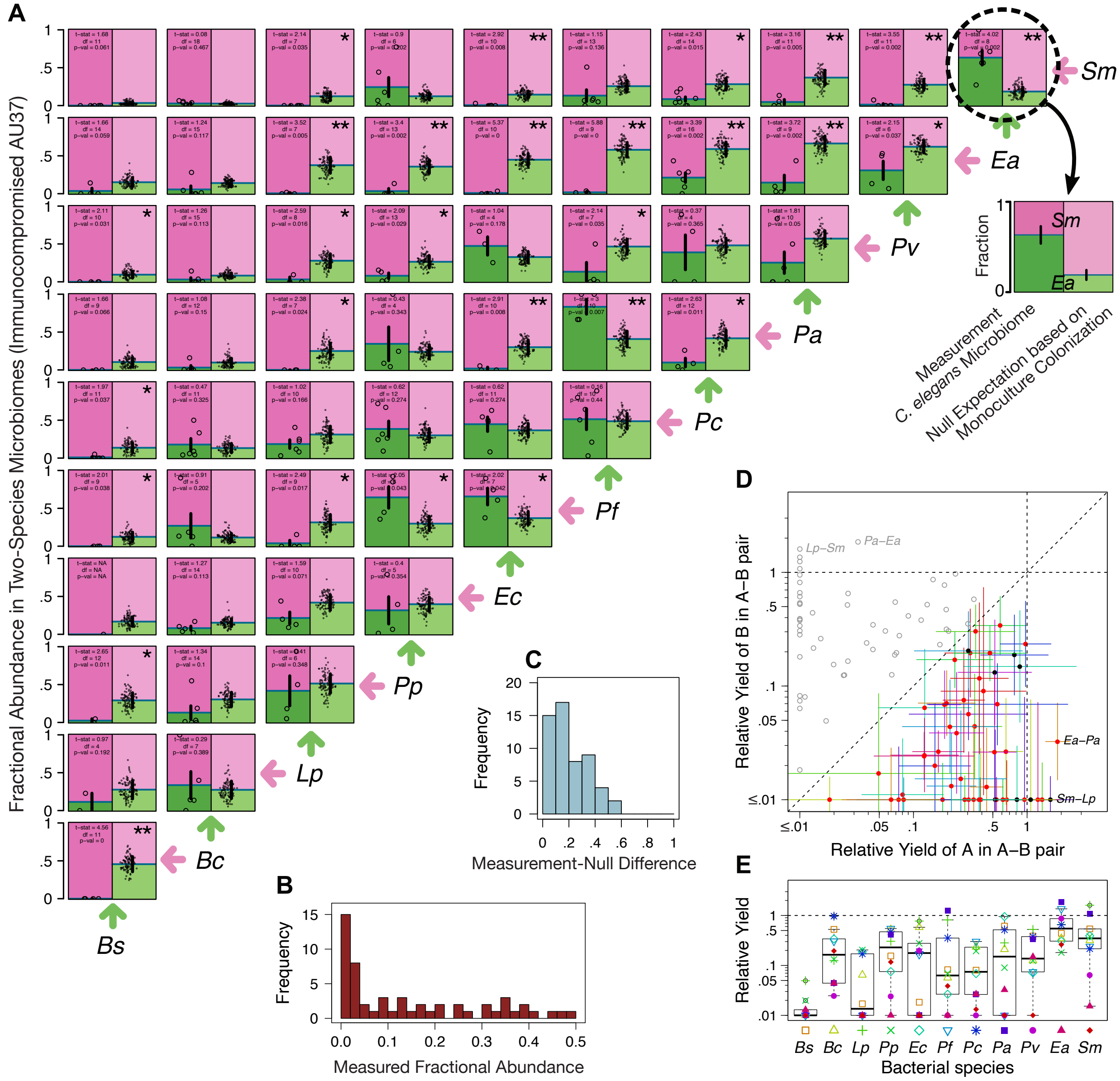

Figure S2. Two-species microbiotas in *C. elegans* intestine contain mostly competitive interactions. (A) LEFT: Fractional abundances of 55 different two-species microbiotas constructed from 11 non-native bacterial species in an immunocompromised worm. Points are the replicates and error bars are the s.e.m. Most conditions had four or more biological replicates. RIGHT: Null expectation based on monoculture colonization. The underlying points, from where the mean null expectation was calculated, are the fractions obtained from combining the monoculture replicates of the two bacterial species, and the error bars are the s.e.m. * and ** represent a statistically significant difference between left and right panels at *p*-values of .05 and .01, respectively (Welch's T-test). Bacterial species are ordered from left to right by their mean fraction across the 10 different two-species microbiotas. **(B)** Histogram of the fractional abundances in the two-species microbiotas show that only a minority of the pairs displayed competitive exclusion (14/55~25%). From a pair of bacteria reaching fractions 57%-43%, only the lower quantity, 43%, was plotted. **(C)** Distribution of the differences between the left and right panels in (A). Across all pairs, the measured fraction deviates from the null expectation by a mean distance of 20%, with a peak at low distance and a long tail corresponding to cases where the

interspecies interactions are particularly important (Figure S2C). **(D)** Relative yields (RY) less than one in two-species microbiotas are indicative of competition. The RY of a bacterial strain is calculated by dividing its population size in a co-culture experiment by its monoculture population size. Each point is the mean RY from 1000 bootstrap replicates sampling simultaneously over the pairwise data and the monoculture data. Error bars are the standard deviation of this bootstrap replicates (or the s.e.m. RY). The points were colored red, or black if the total abundance in co-culture (A+B) is lower or higher than the higher of the monoculture population sizes plus its s.e.m., respectively. Some points were labeled, error bars were colored, and dotted lines were added to improve clarity. The points above the diagonal are equivalent to the points below the diagonal. **(E)** Box plots of the RY of each bacterial species. Partner species is denoted by the colored symbols.

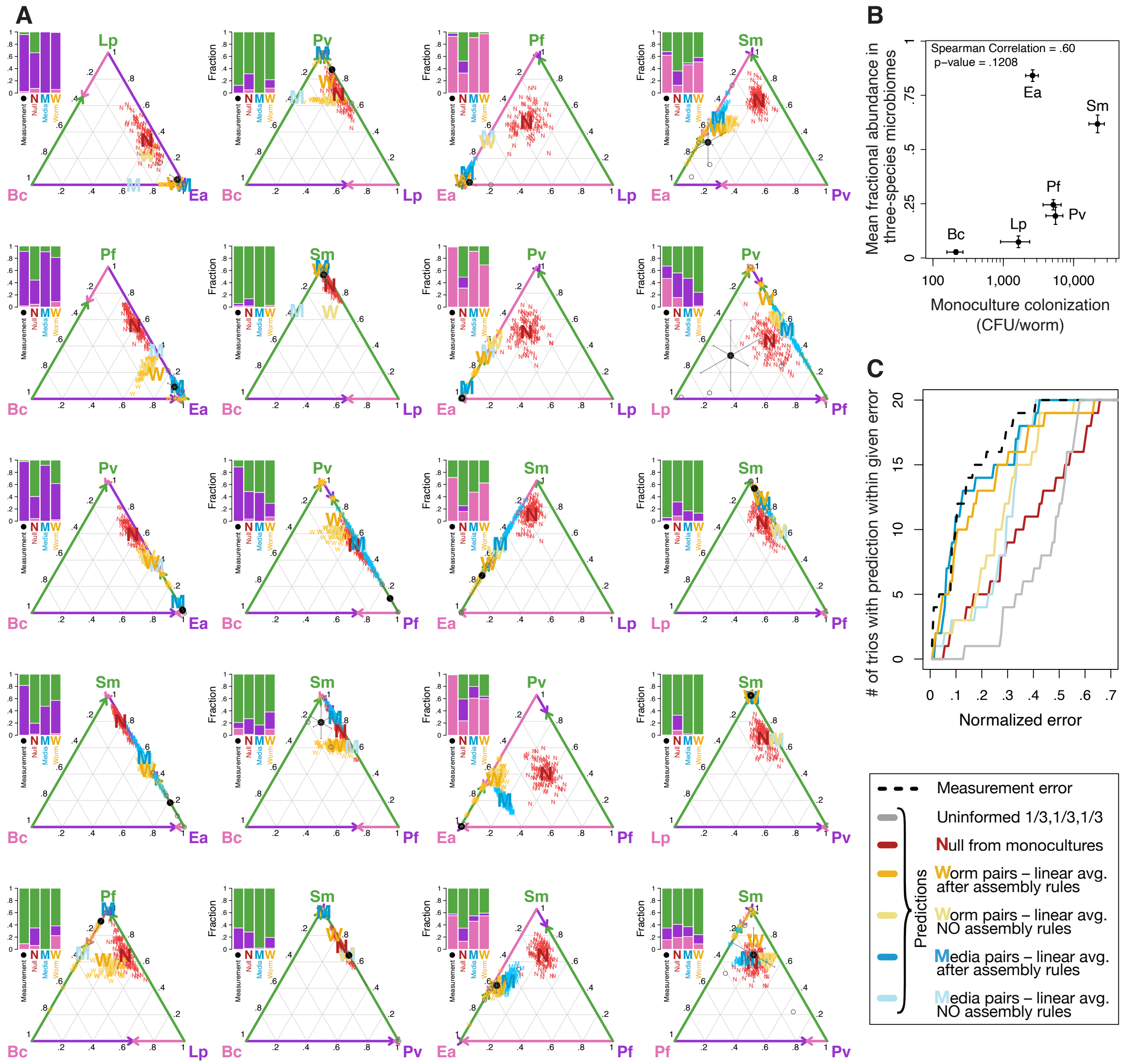

Figure S3. Three-species microbiotas in the *C. elegans* intestine are well predicted based on pairwise outcomes. (A) Each set of bar graphs and the triangle (simplex) to the right displays the measured fractional abundances and predictions of one three-species microbiota. These 20 different three-species microbiotas are all the possible combinations with the species *Bc*, *Lp*, *Pf*, *Pv*, *Ea*, and *Sm*. The edges in the simplexes depict the fractional abundances in the two-species microbiotas. **'N'**: Null expectation based on monocultures, where each bacterial species reaches its population size in monoculture colonization. **'W'**: predictions based on two species microbiotas in worm gut (normalized arithmetic mean with or without *assembly rules*). **'M'**: predictions based on pairwise outcomes *in vitro* liquid media. The error bars on the measurement are the s.e.m. of 4 biological replicates, and the clouds of points around predictions are 100 bootstrap replicates ('N's sampling the monoculture data, and 'W's and 'M's sampling the pairwise data). **(B)** The mean fractional abundance in three-species microbiotas correlates mildly with monoculture population size. Error bars in Y-axis are the propagated error from the s.e.m. of the trios that include the given species. **(C)** Cumulative distribution of the error of the predictions of three-species microbiotas with and without *assembly rules*. The

error of the predictions based on pairs can be reduced by removing a bacterial species from the trio prediction when it cannot survive all co-culture experiments—assembly rules. Errors calculated as the linear distance (L2 norm) between the fractional abundances in the prediction and measurement (normalized by the maximal distance, √2). The dashed line represents the mean distance between the measured mean and the biological replicates of each trio, and it is an upper bound for the error. The gray line is the error of an uninformed prediction of ‘1/3, 1/3, 1/3’, and it is a lower bound for the error.

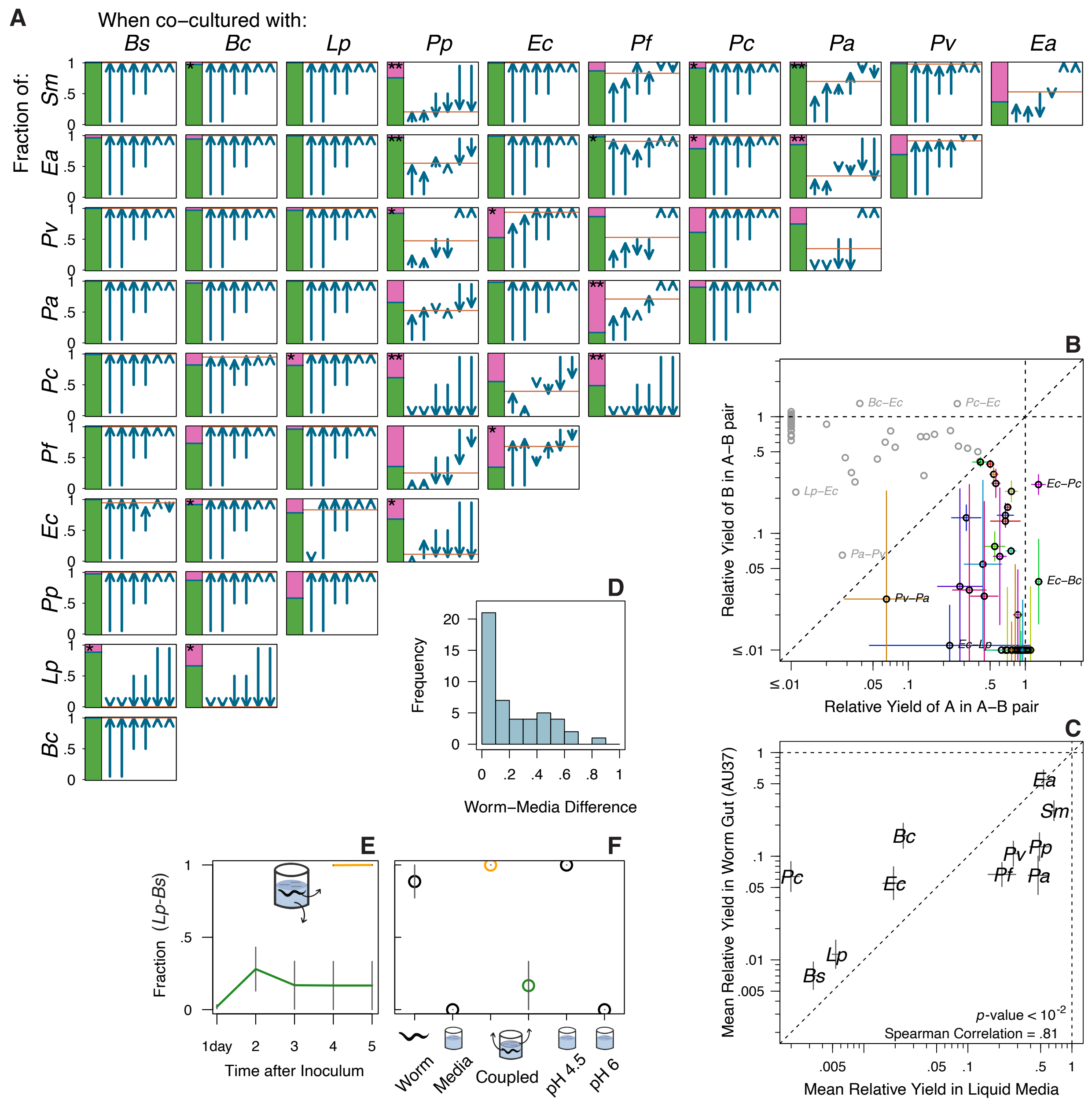

Figure S4. Co-culture experiments *in vitro* liquid media reveal the cases where the worm gut environment determines microbiota composition. (A) Fractional abundances in co-culture experiments in the liquid medium used as feeding substrate (rich medium with peptone, yeast extract, etc., Methods), with each subpanel representing one bacterial pairwise combination, and each arrow representing a replicate. The beginning and the end of each arrow are the starting fraction of each replicate (95-5%, 50-50%, and 5-95%), and the measured fraction after 7 cycles of 100x daily dilution, respectively. Orange lines are the mean across replicates. Green-pink bar-plot on the left of each subpanel is the fractional abundance of the two-species microbiota in AU37 (Figure S2A). * and ** represent a statistically significant difference between worm and liquid media at *p*-values of .05 (19 cases) and .01, respectively (Welch's T-test). **(B)** Low relative yields in liquid media experiments indicate that competition is also the norm in this *in vitro* environment. Each point is the mean RY from 1000 bootstrap replicates sampling simultaneously over the pairwise data and the monoculture data; error bars are the standard deviation of this bootstrap replicates (or the s.e.m. RY). **(C)**

Mean relative yield of each bacteria *in vitro* liquid media and *in vivo* worm gut are correlated. **(D)** Distribution of differences between co-culture experiments *in vitro* liquid media and *in vivo* worm intestine. **(E)** *L. plantarum* and *B. subtilis* reach different fractional abundances *in vivo* worm gut and *in vitro* liquid media on a coupled experiment. **(F)** An acidic version of the media resembling the average pH of the worm intestine (4.5) shifts back the pairwise outcome to a worm-like state; error bars are the s.e.m. of at least 4 replicates.

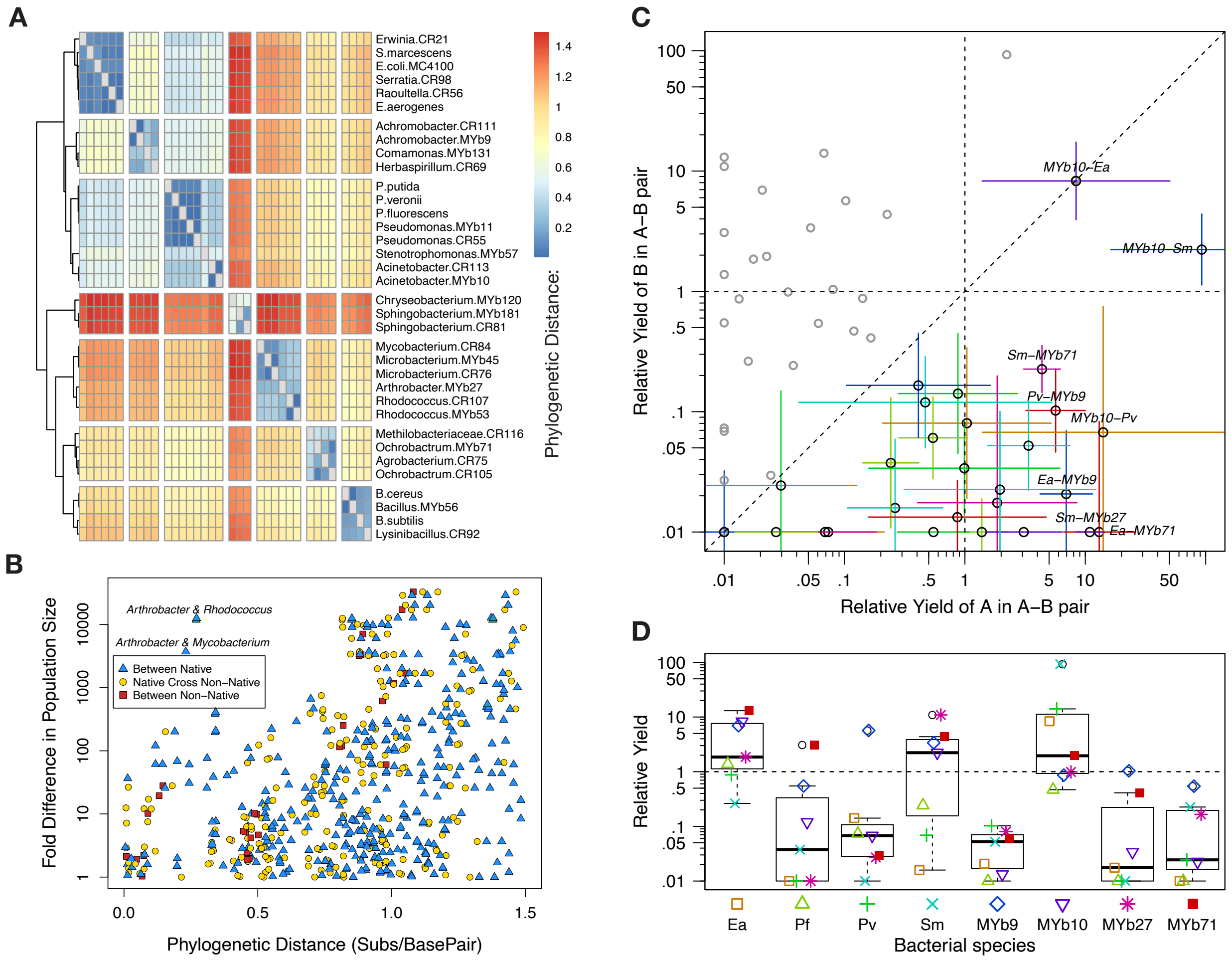

Figure S5. Bacteria isolated from *C. elegans* intestine show more facilitative interactions in two-species microbiotas. (A) Heat-map of phylogenetic distances between native and non-native bacteria in substitutions per base-pair. The distances were calculated directly from the phylogenetic tree (Figure 4A) that was inferred with maximum likelihood from the multiple sequence alignment of the of full-length 16S gene of the bacterial species. Both axis are clustered into 7 bins for ease of interpretation. The phylogenetic tree matches the nomenclature of all bacteria, except for *Stenotrophomonas MYb57*, which fell outside the split between Gamma- and Beta-proteobacteria instead of being inside the Gamma class. More genomic information might be needed to correctly classify MYb57. **(B)** Similar bacteria reach similar population sizes, and as phylogenetic distance increases, the difference in colonization ability tends to increase as well (r_s=.39, p<10^{-15}). Fold differences in population sizes calculated as the ratio of better colonizer over worse colonizer (1 added to each population size to avoid dividing by zero). The phylum Actinobacteria showed the highest variation in colonization (e.g. Arthrobacter and Rhodococcus), which suggests that genomic differences between members of the Actinobacteria might set them apart when colonizing the worm gut. Pseudomonas CR55 is also especially different from its close relatives. **(C)** RY>1 in two-species microbiotas are indicative of facilitation. Each point is the mean RY from 1000 bootstrap replicates sampling simultaneously over the pairwise data and the monoculture data; error bars are the standard deviation of this bootstrap replicates (or the s.e.m. RY). **(D)** Box plots of the RY of each bacterial species. Partner species is denoted by the colored symbols. *E. aerogenes*, *S. marcescens*, and *Acinetobacter MYb10* reach a RY>1 in repeated occasions, especially with native isolates as the co-culture partners.

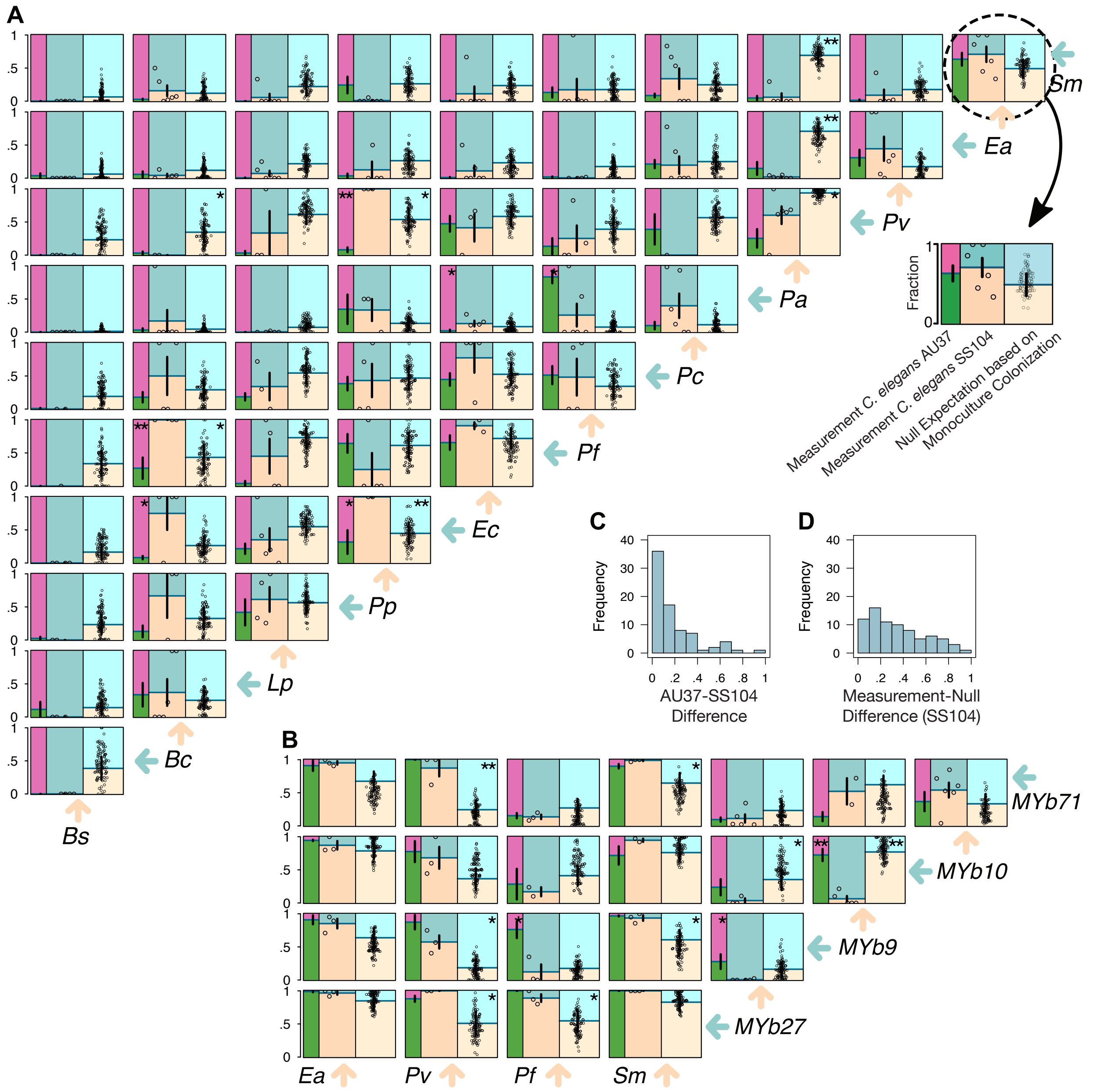

Figure S6. Two-species microbiotas in p38-immunocompetent *C. elegans* reveal the cases where the immune system determines microbiota composition. (A-B) The pink-green bar-plots display the fractional abundances of two-species microbiotas in immunocompromised *C. elegans* AU37 (previously shown in Figures 2A and 4C). The aqua-beige bar-plots display the equivalent two-species microbiotas and the null expectation based on monocultures for immunocompetent worm SS104. * and ** represent a statistically significant difference at *p*-values of .05 and .01, respectively (Welch's T-test). **(C)** 9 out of 77 two-species microbiotas are significantly different between immunocompetent and immunocompromised worms. **(D)** 15 out of 77 two-species microbiotas in immunocompetent worm are significantly different from the null expectation based on monocultures.

Extended Materials and Methods

A synchronous adult population of *C. elegans* was obtained by: 1) Collecting gravid worms from 8-10 plates and extracting eggs with the standard *egg prep* protocol (Stiernagle, 2006). 2) Letting the eggs hatch on M9 Worm Buffer (WB) [3 g KH_2PO_4, 6 g Na_2HPO_4, 5 g NaCl, H_2O to 1 liter. Sterilize by autoclaving and then add 1 ml 1 M $MgSO_4$. *Sigma-Aldrich for all chemicals, unless otherwise specified*] overnight to arrest development of L1 larvae. 3) Transfer larvae to NGM plates with lawns of *E. coli* OP50 and incubate at room temperature for 2 days. The synchronized adult worms were then transferred and kept for 24 hours in 50 ml Falcon tubes (Corning; Corning, NY, USA) with 5 ml S medium, 100 µg/mL gentamicin, and 5X heat-killed *E. coli* OP50 to kill any bacteria inhabiting the intestine, resulting in germ-free worms. S Medium: 1 liter S Basal, 10 ml 1 M potassium citrate pH 6, 10 ml trace metals solution (Teknova), 3 ml 1 M $CaCl_2$, 3 ml 1 M $MgSO_4$. Add components using sterile technique; do not autoclave. S Basal: 5.85 g NaCl, 1 g K_2HPO_4, 6 g KH_2PO_4, H_2O to 1 liter. Sterilize by autoclaving and then add 1 ml cholesterol (5 mg/ml in ethanol). Heat-killed OP50 was prepared by concentrating 50x a saturated culture of *E. coli* OP50, and it was used to trigger feeding behavior in the worms. The adult worms were washed via sucrose flotation to remove debris before bacterial colonization.

The natural microbiota strains of *C. elegans* were isolated by growing *C. elegans* on different types of rotten organic material, followed by washing and sterilizing the worms on the outside, grinding the worms, and plating the resulting bacterial suspension on agar plates. Different types of compost and rotten fruits and vegetables were fed to the worms. Rotten apples were directly collected from the outside. Other fruits like apples, celery, almonds and parsnip were placed on local soil from Boston, MA in a household plastic box (Sterilite) with semi-open lid and incubated at room temperature until the fruits were strongly decayed (~3 weeks). The compost samples were taken from two local compost piles in Boston, MA, that mostly contained kitchen and garden waste. Some amount of PBS and glass beads were added to the samples. The samples were homogenized by vortexing at high speeds. The resulting solution was filtered (Millex-SV 5 µm, MerckMillipore) to remove bigger particles. The resulting emulsion was spread on S media agar plates without citrate. *C. elegans* N2 were first grown on OP50 lawn on NGM plates, sterilized with antibiotics (5 ml S medium, 100 µg/mL gentamicin, 5X heat-killed E. coli OP50) and added to the plates with rotten organic material for approximately one week. After that time, the worms were washed off the plates with M9 + 0.1% Tx. The worms were washed twice with 1 mL WB + 0.1% Tx (centrifugation at 2K RCF, 10s). Afterwards the worms were re-suspended in 1 mL ice-cold WB + 0.1% Tx and incubated on ice for 10 mins. 2 µL bleach (Clorox)

were added and the worms were incubated for 6 mins on ice. Afterwards the worms were washed 3x with ice-cold WB + 0.1% Tx. Single worms were transferred into 0.6 mL reaction tubes (Eppendorf) and ground with a motorized pestle (Kimble Kontes Pellet Pestle, Fisher Scientific) for at least 1 min. The resulting solution was plated onto a tryptic soy broth (Teknova) agar plate (2% agar, 150mm petri dish). From the resulting colonies, physiological unique colonies were picked, and streaked-out again on tryptic soy broth agar to isolate the strain. Finally, the bacteria were grown in tryptic soy broth at 30°C and stored as glycerol stocks. The species identity was analyzed by 16S Sanger sequencing (Genewiz; South Plainfield, NJ, USA).

The hierarchy score of a matrix with fractional abundances is calculated by: 1) Ordering its rows and columns ascendingly based on the mean fractional abundance; and 2) Taking the mean value of the half-matrix under the diagonal. In a perfectly hierarchical matrix, each competitor will drive to extinction every other species with a lower rank, reaching a hierarchy score of 1. Random matrices were generated to calculate the significance of the observed high hierarchy score. We conserved the distribution of fractional abundances by: 1) Sampling with replacement 55 values of the original matrix; 2) Assigning these random fractional abundances to the lower triangle of a new matrix; and 3) Assigning to the upper triangle of the matrix the values of 1-transpose. For each random matrix generated, a new hierarchy score is calculated as previously described.
